# Oculomotor Paralysis, Postorbital Pain, and Hypopituitarism as First Presentations of Metastatic Gastric Cancer in the Pituitary Flourished by Internal Carotid Aneurysm: A Case Report

**DOI:** 10.1097/MD.0000000000002317

**Published:** 2015-12-18

**Authors:** Chuanwei Yang, Hongqiang Zhang, Shiqiang Zhang, Ling Liu, Binbin Ma, Jiacheng Lou, Xiaorui Sun, Bo Zhang

**Affiliations:** From the Department of Neurosurgery of the Second Affiliated Hospital of Dalian Medical University (CY, HZ, SZ, BM, JL, XS, BZ); and Institute of Cancer Stem Cell (LL), Dalian Medical University, Dalian, PR China.

## Abstract

Metastatic gastric cancer in the pituitary (MGCP) is rare. Few are known on the clinical and radiological characteristics of MGCP. To date, the coexistence of metastatic pituitary tumors and intracranial aneurysms has not been reported in literatures.

We present a case of MGCP with internal carotid aneurysm in a 57-year-old woman, who presented with oculomotor paralysis, postorbital pain, and hypopituitarism as onset symptoms. The patient had a history of the surgical removal of gastric cancer. Magnetic resonance imaging and single-photon emission computed tomography revealed a recurrent sellar mass with intracranial and multiple bone metastases. The patient underwent subtotal removal of the tumor, followed by conformal radiotherapy and chemotherapy. Ten months after surgery, the patient died due to deterioration of her overall condition.

We also reviewed and analyzed the clinical data, imaging features, and treatment methods of additional 4 cases with MGCP, which were reported in literatures. This study provides important clinical information for the diagnosis and treatment of MGCP.

## INTRODUCTION

Metastasis in the pituitary (MP) is a rare cancer-associated complication, accounting for <1% of all sellar or parasellar tumors and ∼5.1% of all metastatic brain tumors.^1^ Although MPs can be seen in young people,^2^ they usually affect elderly people^3^ without gender predominance.^4^ Diabetes insipidus and hypopituitarism are the most common symptoms of tumors metastatic to the pituitary. The pituitary gland may be the only metastatic site of a tumor, and MP may cause the first clinical presentation of tumors with multiple metastases. Breast cancer and lung cancer are the most common primary tumors metastatic to the pituitary gland in women and men, respectively.^5,6^ MP from gastric cancer is rarely reported in literatures.^4,7,8^ Gastric cancer commonly metastasizes via the lymph node, peritoneum, blood, or bone marrow, and common metastatic sites are the liver, peritoneum, lung, and bone. Intracranial aneurysm is found in only 0.3% of brain tumor patients,^9^ and a coexisting aneurysm can trigger or exacerbate the disease course of tumors.^10,11^ Tumors metastatic to the pituitary gland and parasellar aneurysms are usually misdiagnosed as pituitary adenoma. To date, the coexistence of MP and intracranial aneurysms has not been reported in literatures. Therefore, a few information is available regarding the clinical and radiological characteristics of metastatic gastric cancer in the pituitary (MGCP) coexisting with internal carotid aneurysm (ICA).

In this study, we present the first reported case of MGCP with a left ICA in the siphon segment in a 57-year-old woman, presenting with oculomotor paralysis, postorbital pain, and hypopituitarism. In addition, we reviewed 4 other known cases with MGCP and summarize the clinical manifestations and imaging characteristics of MGCP. Our study provides important clinical information for diagnosis and management of MGCP.

## CASE REPORT

In September 2014, a 57-year-old woman visited our hospital presenting with diplopia, paroxysmal headache, and postorbital pain without obvious causes. The patient had right severe ptosis 1 week later. Four years earlier, the patient underwent subtotal gastrectomy for gastric antrum carcinoma followed by 1 cycle of FOLFOX (Oxaliplatin+leucovorin+5-FU) and 4 cycles of XELOX (Xeloda+oxaliplatin) chemotherapy. She had no family history of gastric cancer.

**FIGURE 1:**
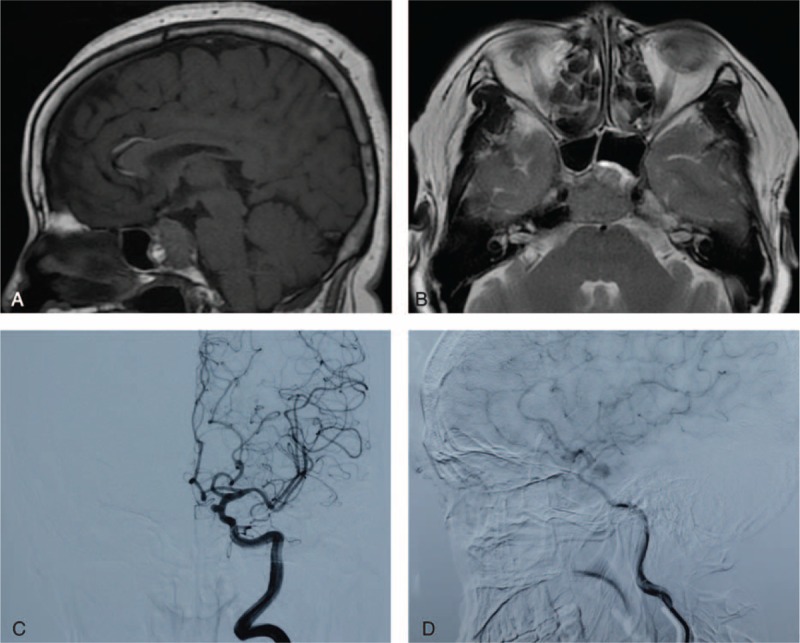
Radiological findings: sagittal T1-WI (A) and axial T2-WI (B) of MRI revealed the pituitary mass with suprasellar extension that mimicked pituitary adenoma. DSA revealed the ICA (C) and blood vessels supplying the sellar mass (D). DSA = digital subtraction angiography; ICA = internal carotid aneurysm; MRI = magnetic resonance imaging.

On physical examination, the patient had right oculomotor paralysis with right eyelid drooping and was unable to perform the inward, upward, and downward movement of the right eyeball. She had diplopia without nystagmus. The right pupil was dilated with blunt pupillary reflex. Visual field and visual acuity of both eyes were normal. Computer tomography (CT) scan of the orbital, chest, and abdomen were unremarkable. Brain CT revealed a destroyed occipital base and dorsum sellae surrounded by soft-tissue density shadows. Brain magnetic resonance imaging (MRI) revealed a round mass of 25 mm in diameter in the enlarged sellae (Figure 1A and B). T1-weighted images (T1-WI) revealed a round mass with isointense and hyperintense signals, whereas T2-weighted images (T2-WI) revealed a mass with a hyperintense signal with heterogeneous enhancement after Gadolinium-DTPA injection. The sellae was elevated with a left displacement of the pituitary stalk and a left shift of the bilateral optic chiasma and cavernous sinus. The patient was diagnosed with a giant pituitary adenoma. Laboratory findings revealed reduced levels of free triiodothyronine (FT3), free thyroxine (FT4), and cortisol (Table 1). CT angiography (CTA) of the intracranial artery revealed an ICA of 3.0 × 3.0 × 3.4 mm at the inner edge of the siphon segment and the aplastic left posterior cerebral artery. Digital subtraction angiography (DSA) revealed that the blood supply to the pituitary was from the meningeal pituitary branch of the right intracranial artery and the aneurysm (Figure 1C and D).

**TABLE 1:**
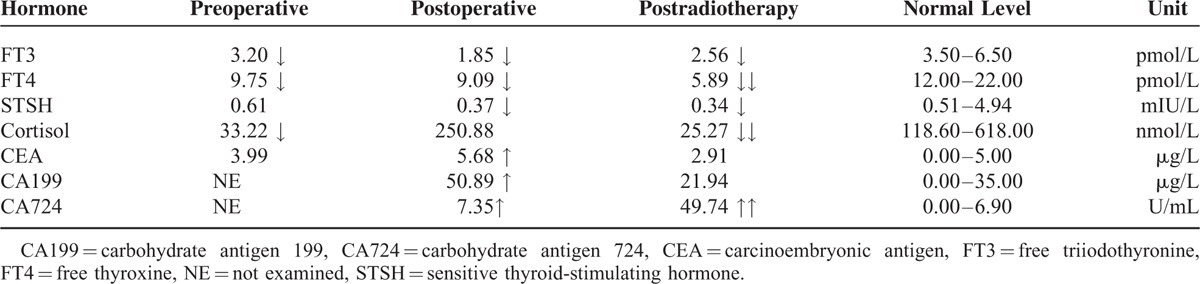
Laboratory Findings

**FIGURE 2:**
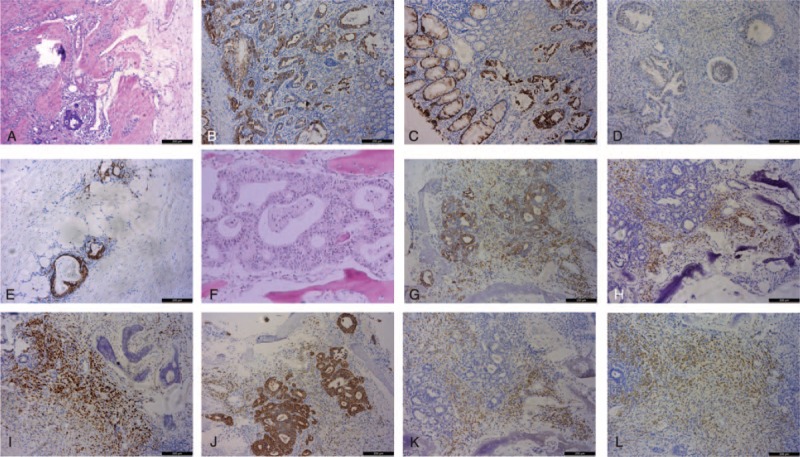
Hematoxylin and eosin (H&E) staining (A and F, 100×) and immunohistochemical staining (B, C, D, E, G, H, I, J, K, and L, 100×) of the primary gastric carcinoma (A–E) and MP (F–L). Primary gastric cancer was ulcerative gastric adenocarcinoma with poor to moderate differentiation, histological grade II, and pT4aN0M0 stage. The tumor was immunopositive for CK7 (B), CK20 (C), CDX-2 (D), and villin (E). MGCP revealed poorly differentiated atypia adenocarcinoma cells (F), was immunopositive for CK7 (G), CK20 (H), CDX-2 (I), and villin (J), and was also immunopositive for CgA (K) and Syn (L). H&E = hematoxylin and eosin; MGCP = metastatic gastric cancer in the pituitary; MP = metastasis in the pituitary.

The patient underwent transnasal transsphenoidal surgery to remove the tumor. The tumor was red and solid with rich blood supply, and infiltrated into the clivus with close adhesion to the bone. The upper left of the tumor surrounded the artery aneurysm. The tumor was subtotally resected in pieces. The aneurysm remained unprocessed due to its small size and wide neck. Postoperative pathological examination revealed that the tumor was adenocarcinoma resembling primary gastric carcinoma (Figure 2A and F). Optic tissues and pituitary tissues were distributed among the adenocarcinoma tissue. The tumor was immunopositive for gastric cancer markers such as CK7, CK20, CDX-2 and villin, and neuroendocrine tumor markers such as CgA and Syn (Figure 2). The Ki67 index was 60%. The patient was diagnosised with MGCP. After surgery, postorbital pain and headache disappeared, and no relief was seen in other symptoms and signs. The patient was treated with intravenous injection of hydrocortisone (100 mg, q.d.) and oral administration of levothyroxine sodium tablets (50 μg, b.i.d.). Laboratory findings revealed that FT3, FT4, and sensitive thyroid-stimulating hormone (STSH) serum levels further decreased, whereas carcinoembryonic antigen (CEA), CA199, and CA724 serum levels were elevated.

**FIGURE 3:**
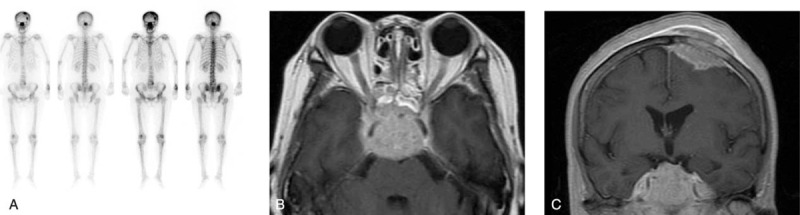
SPECT and MRI revealed that the pituitary mass recurred with multiple bone metastasis and intracranial metastasis after surgery. (A) SPECT performed 1 month after surgery reveals the tumor metastasis in the skull, skull base, 3rd thoracic vertebra, and the right sacroiliac joint. MRI performed 3 months after surgery reveals the pituitary mass (B) and left frontoparietal mass (C) that recurred. MRI = magnetic resonance imaging, SPECT = single-photon emission computed tomography.

One month after the surgery, the patient complained of bone pain, headache, dizziness, fatigue, and nausea with vomiting. MRI revealed a giant round sellar mass of 28 mm in diameter with an unclear boundary. The tumor was isointense or hyperintense on T1-WI, and isointense or hypointense on T2-WI with heterogeneous enhancement. SPECT revealed that multiple bone metastases occurred in the skull, skull base, 3rd thoracic vertebra, and the right sacroiliac joint (Figure 3). Recurrence of the sellar mass and multiple bone metastases was considered. One month later, MRI indicated that the sellar mass increased and infiltrated into the bilateral optical nerves. The patient received intensity-modulated conformal radiotherapy with DT5040cGy in 28 fractions in the brain lesion area. After radiotherapy, the patient developed bilateral ptosis, diplopia, and occasional vomiting. FT3, FT4, STSH, and cortisol levels sharply decreased, suggesting the pituitary crisis precursor. The patient was immediately treated with intravenous injection of hydrocortisone (100 mg, b.i.d.) and oral administration of levothyroxine sodium tablets (50 μg, q.d.). One week after radiotherapy, symptoms improved and the left ptosis was relieved. The patient received oral administration of prednisone (5 mg at 8:00 am and 2.5 mg at 3:00 am) and levothyroxine sodium tablets (50 μg, q.d.) daily. One month after the radiotherapy, MRI revealed that the size of the sellar mass did not change. A soft-tissue mass of 38 × 42 mm in size was found in the left frontoparietal area with a slightly hypointense signal on T1WI and T2WI, which was enhanced after contrast injection (Figure 3). SPECT revealed that bone metastasis was aggravated. The patient received intravenous injection of zoledronate (5 mg) twice in 2 weeks, followed by 2 cycles of chemotherapy with oxaliplatin (200 mg on the 1st day), and gimeracil and oteracil potassium capsules (60 mg, b.i.d., from the 1st day to the 14th day). One month after chemotherapy, MRI revealed that the size of the tumor mass in the sellae and left frontoparietal area became larger, and the brain midline slightly shifted to the left. FT4, STSH, cortisol, CEA, and CA199 levels returned to normal, whereas the FT3 level (3.23 pmol/L) was slightly lower than normal. The CA724 level increased to 49.74 U/mL. The patient had stable symptoms and was discharged from the hospital. At the latest follow-up (in August, 2015), the patient died due to deterioration of her overall condition.

This study was approved by the Second Affiliated Hospital of Dalian Medical University, China. The patient provided informed consent.

## DISCUSSION

Carcinomas originate from almost every tissue and can metastasize to the pituitary.^12^ Metastatic tumor cells can reach the sellae via hematogenous spreading, extension from juxtasellar tissues and the skull base, and dissemination through lymphatic microvessels.^13^ The most common tumors metastatic to the pituitary are breast cancer and lung cancer, followed by prostate cancer, intestinal cancer, and liver cancer. Although metastasis from gastric cancer such as adenocarcinomas in the upper and lower parts of the stomach is not uncommon,^14,15^ metastasis of gastric cancer to the pituitary gland is rare. To date, there are only 4 cases of MGCP in literatures^16–19^ (Table 2). In this study, we report the fifth case of MGCP, which is unique for its coexistence with ICA. Neuroimaging and intraoperative findings revealed that MGCP was supplied by aneurysmal blood vessels. The abnormal expansion of the artery wall and blood flow vortex of aneurysmal vessels may increase the dwelling and thriving of circulating tumor cells, and thus, promote tumor metastasis. In addition, the surrounding tumor weakens the artery wall, which may promote its abnormal expansion into the artery. Furthermore, the hormone-rich microenvironment of the pituitary may attract tumor cells, promoting the development of metastasis.^7^

**TABLE 2:**
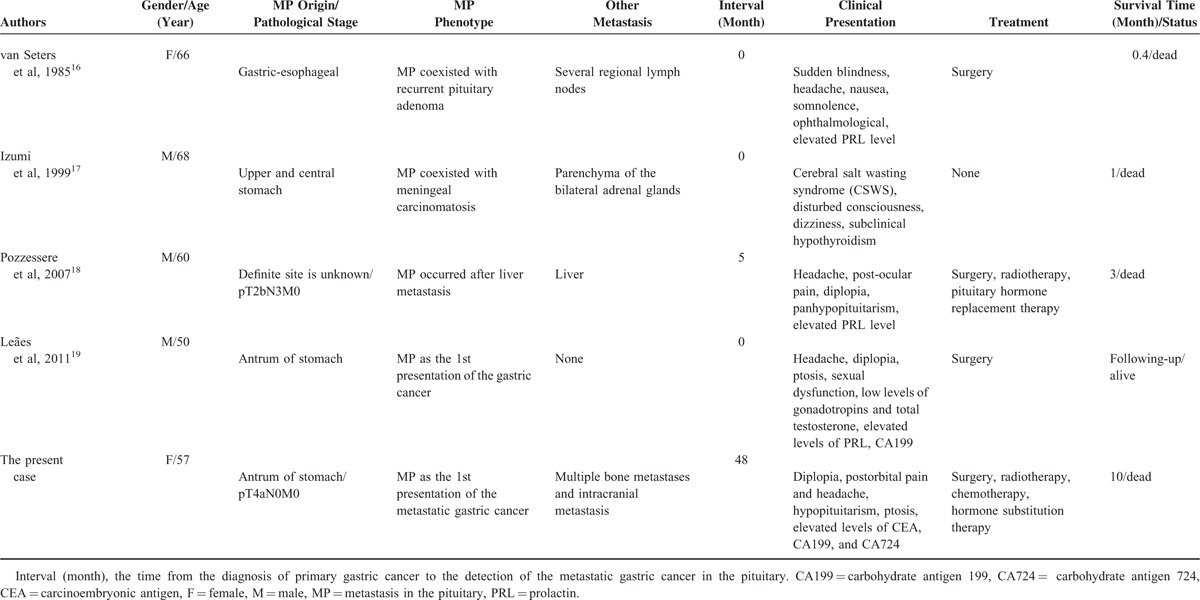
Summary of Patients With MP From Gastric Adenocarcinoma

Gastric cancer from the upper and lower stomach is prone to metastasis to the pituitary (Table 2). Among the 5 cases with MGCP, diplopia, headache, and hypopituitarism are the most frequent symptoms, which occurred in 3, 3, and 2 cases, respectively. These symptoms are possibly caused by the invasion of prehypophysis and compression of surrounding brain tissues. Diplopia is rarely associated with invasive pituitary adenomas in the absence of pituitary apoplexy.^20^ Therefore, if diplopia is presented in patients with a history of gastric cancer, a metastatic lesion within the sellae should be considered even long after the primary tumor is totally-resected. Diabetes insipidus is the most common symptom of MP and often presents in metastatic or primary lesions in the posterior pituitary or impaired pituitary stalk after sellae surgery.^21,22^ Thus, diplopia and diabetes insipidus may be helpful in the differential diagnosis of lesions in the anterior pituitary or posterior pituitary. In 3 cases, elevated levels of PRL are observed. However, the level of PRL remained normal in the present case. In addition, our patient had postorbital pain, which was possibly caused by postorbital dura irritation by the giant sellar mass or ICA. In our case, with the development of recurrent MP and extra-sellar metastasis, the patient also presented with right ptosis, a decline of right visual acuity, fatigue, transient nausea, vomiting, and tinnitus.

It has been reported that the simultaneous elevated levels of perioperative CEA, CA199, and CA724 suggest the metastasis and poor prognosis of gastric cancer.^23,24^ CA724 is the most sensitive serum tumor biomarker for gastric cancer, which can be used for predicting the sensitivity to chemotherapy.^25^ In combination with CEA, CA125, and CAl99, CA724 can improve sensitivity for the early diagnosis of gastric cancer.^26^ In the present case, we found that the level of CEA and CA199 increased with the development of MP and decreased to normal after radiotherapy. CA724 levels continued to increase with the progression of MP. Therefore, CEA, CA199, and CA724 are useful markers for the early diagnosis and follow-up examination of gastric cancer.

Generally, it is advisable to surgically remove coexistent brain tumors and aneurysms. However, mortality associated with surgical treatment is very high. It has been reported that the mortality rate is ∼38% in patients receiving surgical removal of brain tumors or aneurysms alone, or both.^9,27^ For metastatic tumors, total removal is not feasible due to the high vascularity of the tumor, package of the internal carotid artery and cranial nerves, and local invasion into the surrounding bones and cavernous sinus, thus, making it easy to recur. In most cases, local radiotherapy and chemotherapy can improve the quality of life of patients, but not the survival of patients with MP.^1^ Morita et al^7^ reported an improvement in survival with multimodality treatments including surgery, radiotherapy, or chemotherapy. Among the 5 cases with MP, patients who were diagnosed early had the pituitary gland as the solitary metastatic site, or treated with surgery, radiotherapy, or chemotherapy tended to have better survival (Table 2).

Neuroimaging is helpful for differential diagnosis between MP and pituitary lesions such as pituitary macroadenoma, pituitary apoplexy, cystic lesions, and chronic inflammation. MRI is not specific and usually shows isointense or hyperintense signals on T1-WI and isointense or hypointense signals on T2-WI.^3,7^ CT usually reveals a hyperdense or isodense mass. Although positron emission tomography (PET) is more sensitive with sharper images for differential diagnosis than SPECT, it is not routinely used due to its high cost.^28^ SPECT can detect multiple metastases early^29^ and is recommended for differential diagnosis. Although neuroimaging findings are helpful for differential diagnosis, these findings cannot reliably distinguish MP from adenoma. As clinical syndromes are distinctive between MP and adenoma, clinical manifestations are important for differential diagnosis.^30^ Therefore, these following features favor the diagnosis of MP: positive history of cancer, fast growth of the tumor, presence of diplopia or diabetes insipidus, rapid development of anterior pituitary hormone deficiencies, high level of cancer biomarkers, positive findings in SPECT/PET examinations, and atypical appearance of the tumor. Definitely, pathological diagnosis is conclusive.

In summary, we describe a rare case, in which a patient had a sellar mass close to the left internal carotid artery aneurysm. MP should be considered in patients with a history of gastric cancer and had rapidly aggravated diplopia, postorbital pain, headache, and hypopituitarism. CEA, CA199, and CA724 serum levels may be useful markers for monitoring disease progression. Combined treatments including surgical treatment, radiotherapy, chemotherapy, and hormone replacement therapy may be important for improving the survival of patients with MP.
